# Influence of Construction Material Type on the Dynamic Response of Low-Rise Buildings

**DOI:** 10.3390/ma19143125

**Published:** 2026-07-21

**Authors:** Maciej Zajac, Krystyna Kuzniar, Tadeusz Tatara

**Affiliations:** 1 Faculty of Civil Engineering, Cracow University of Technology, ul. Warszawska 24, 31-155 Krakow, Poland; tadeusz.tatara@pk.edu.pl; 2 Institute of Technology, University of the National Education Commission, ul. Podchorazych 2, 30-084 Krakow, Poland; krystyna.kuzniar@uken.krakow.pl

**Keywords:** construction materials, material stiffness, low-rise building, dynamic response, 3D numerical studies, mine-induced vibrations

## Abstract

**Highlights:**

- Material stiffness governs the dynamic response of low-rise buildings.
- Validated 3D FEM model quantifies responses to real mine tremors.
- Dominant response frequencies depend on wall material and estimation point location.
- Dynamic response of a low-rise building depends on the wall material and the choice of estimation point location.
- Low-stiffness materials amplify vibrations and damage risk.

**Abstract:**

This study presents a numerical investigation of the dynamic response of a typical low-rise building subjected to recorded mine-induced vibrations, with particular emphasis on the role of construction material type. Several structural variants consistent with the real building configuration were analysed using a validated three-dimensional finite element (3D FEM) model. Seven load-bearing wall materials were considered, including reinforced concrete, lightweight concrete, cellular concrete, standard brick, and selected sand–lime bricks. Dynamic responses were evaluated in terms of displacements and accelerations, including time histories along the building axis, peak component values, resultant responses, and Fourier spectra. The results clearly demonstrate that material properties—especially stiffness—govern the dynamic behaviour of the structure. Low-stiffness materials, such as cellular concrete, significantly amplify both acceleration and displacement responses, increasing susceptibility to vibration-induced effects. Moreover, the dominant vibration frequencies were found to vary depending on the wall material, which directly affects resonance conditions and potential damage risk. A notable sensitivity of the calculated response to the choice of numerical evaluation point within the structure was also observed. The findings highlight the critical importance of material selection in controlling the dynamic performance of low-rise buildings exposed to mining-induced vibrations and provide practical guidance for mitigating paraseismic effects in engineering design.

## 1. Introduction

Mining-induced seismic events, caused by the exploitation of underground mineral deposits, pose a significant threat to the structural integrity and safety of buildings within mining-affected zones. These events differ markedly from natural tectonic earthquakes in key aspects such as frequency content, duration, and spatial distribution [1,2]. Such differences necessitate the development and application of specialised numerical methods tailored to the unique features of mining tremors. Conventional seismic design approaches—usually based on probabilistic models of tectonic activity—may not sufficiently capture the localised and repetitive nature of mining-induced ground motions. Consequently, there is a growing need for practical, site-specific methodologies that can reliably predict the structural response of buildings subjected to these dynamic loads. The dynamic response of structures to mining-induced ground motions has become a key area of research, especially in regions with extensive underground mining operations. Low-rise buildings, which make up a large portion of the built environment in these areas, are particularly vulnerable to vibrations generated by mining activities. Those buildings are often subjected to dynamic excitations caused by mining-induced seismic events [3,4,5,6]. Despite the problem with active seismicity in mining areas worldwide, most of the published studies concerning low-rise buildings are limited to earthquake-induced building vibrations [7,8,9,10,11]. Although typically of lower magnitude than natural earthquakes, mine-induced vibrations can still cause structural damage, impair serviceability, and contribute to long-term degradation of low-rise building performance. Therefore, understanding and accurately estimating the dynamic response of such structures is essential to ensure their safety, resilience, and longevity. The dynamic response of such structures results from a complex interaction of factors, including the characteristics of seismic excitation, the mechanical properties of building materials, the structural dynamic attributes, and the soil–structure interaction.

In some cases, an approximate assessment of the harmfulness of mining-induced vibrations can be performed using procedures specified in standards (e.g., the Polish standard [12]) or so-called mining scales developed for a given mining region (e.g., the Mining Seismic Intensity Scale MSIIS-2022 [13]). However, in recent years, numerical methods have become indispensable tools for evaluating the dynamic behaviour of buildings subjected to seismic-type loads. For instance, the application of artificial neural networks (ANNs) to analyse the dynamic behaviour of structures affected by mining-related and other ground motions was presented [14,15,16,17]. The results demonstrate that ANN-based approaches offer efficient and sufficiently accurate analyses of structural dynamics, representing a promising alternative to conventional numerical techniques, also in the context of mining-induced seismicity. Nevertheless, the use of techniques such as the finite element method (FEM) still dominates among numerical methods in the analysis of the effects of seismic-type vibrations on buildings, including mine-induced vibrations [3,4,5,18,19,20,21,22]. FEM has proven particularly effective in simulating structural responses to ground motion, enabling engineers to assess potential damage and refine design strategies. However, the accuracy and reliability of these models are highly dependent on the quality of input data, including, among others, ground motion records, soil–structure interaction parameters, and structural detailing. Additionally, for practical reasons, the selection of the FEM model mesh nodes whose calculated dynamic response will be considered for assessing the harmfulness of vibrations to the building may also be important. Unfortunately, there is a lack of work on this topic.

In this paper, a comprehensive numerical analysis of a typical low-rise office building subjected to mining-induced vibrations is presented. This study investigates the building response to actual paraseismic excitations resulting from underground mining activities. By conducting direct free-field vibration measurements and analysing the influence of material properties and impact of the choice of the place of numerical estimation on the calculated building dynamic mine-induced response, it is possible to capture the authentic dynamic behaviour of structures subjected to mining-induced ground motions. Several variants of low-rise buildings with structural systems the same as the construction of the actual building were analysed. Seven different load-bearing wall materials were considered, including standard reinforced concrete, lightweight reinforced concrete, cellular concrete, standard brick, and three types of sand–lime bricks. The building was modelled using a validated three-dimensional (3D) finite element method (FEM) model, and its response to three recorded mining tremors of varying energy levels was analysed. Both dynamic responses in the form of displacements and accelerations were analysed. For both displacements and accelerations, time histories in directions parallel to the building axis, peak values of vibration components, resultant vibrations, and Fourier spectra were taken into account.

This study offers several novel insights into the dynamic behaviour of low-rise buildings subjected to mining-induced vibrations. The key contributions are summarised as follows:

1. Identification of the influence of construction material properties on low-rise building dynamic response;
2. Comparison of the numerically obtained low-rise building dynamic mine-induced responses calculated for different point locations in the building;
3. Analysis of the variation in calculated building response dominant frequencies with wall material as well as the choice of computing point location;
4. Investigation of the effect of mining energy levels on low-rise building dynamic response depending on the wall-bearing material variant and the choice of computing point location;
5. Presentation of a 3D FEM model validated with real mine-induced events, intended to determine the responses of buildings to seismic-type impacts.

This article aims to bridge the gap between theoretical modelling and practical application by offering insights into the numerical estimation of mining-induced dynamic responses in low-rise buildings. Drawing on case studies, field measurements, and simulation results, it explores key factors influencing structural response, including response location selection, material properties, and proximity to mining sources. By focusing on practical aspects of numerical estimation, this study may support civil engineers, researchers, and decision-makers in developing more effective mitigation strategies for mining-related structural risks.

## 2. Analysed Building Vibrations Induced by Mining Tremors

### 2.1. Variants of the Building

**Figure 1:**
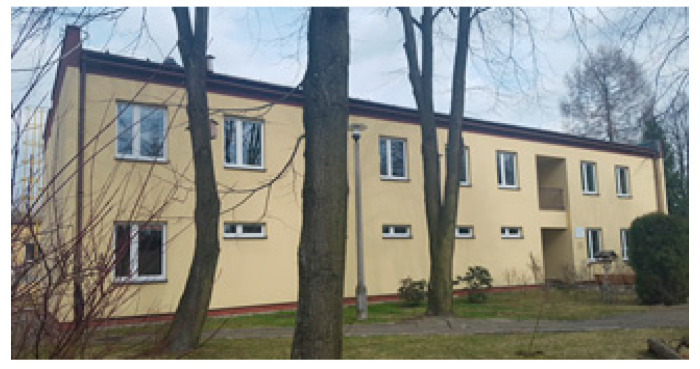
A view of the low-rise building.

In order to numerically estimate mine-induced dynamic responses in low-rise buildings, the construction of the actual building was taken into consideration. This is an administrative (office) two-storey building, which is commonly found and typical for surface infrastructure in mining regions. A photo of the actual building is shown in Figure 1.

The building sub-soil consists of a soil layer, and below layers of medium sand and fine sand with inclusions of yellow dust. The building does not have a basement. It is founded directly on the ground using concrete strip foundations. The foundation depth is 1.4 m. Vertical bearing elements (load-bearing walls) of the considered building construction create a mixed transverse–longitudinal system. The horizontal structural elements include reinforced-concrete floor slabs, while the roof is supported on knee walls.

**Figure 2:**
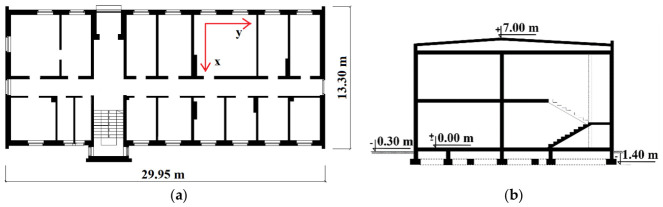
Floor plan view (**a**) and vertical cross-section view (**b**) of the typical, actual office building.

The floor plan and vertical cross-section corresponding to the representative low-rise building are schematically presented in Figure 2. Additionally, the building dimensions and the coordinate axes x and y, corresponding to the transverse and longitudinal directions, respectively, are shown in Figure 2.

The analysed building is representative of low-rise structures commonly found in mining areas, including administrative and infrastructure facilities, which often exhibit similar construction solutions and are particularly exposed to anthropogenic seismic effects.

Several variants of low-rise buildings were analysed. Structural systems of all of the considered items are the same as the construction of the actual building (see Figure 1 and Figure 2). Reinforced-concrete ceilings and prefabricated roof panels are applied in the case of all of the variants and are also the same as those used in the actual building. The main difference concerns the load-bearing walls of the analysed building variants.

Seven various construction wall materials were taken into consideration and, as a result, seven building variants were obtained. They were designated as M1 to M7, correspondingly to the applied load-bearing wall material. Names and denotations of the seven construction materials, and their most important parameters, are collected in Table 1.

### 2.2. Sources of Building Vibrations

Mining tremors occurring in the Upper Silesian Coalfield (USC) area in Poland were used as the sources of low-rise building vibrations. The USC region is an area affected by very intensive mining-induced seismic activity.

Free-field vibrations were measured in situ at a surface seismological station next to the building. Registration of free-field vibrations was performed using monitoring equipment.

**Figure 3:**
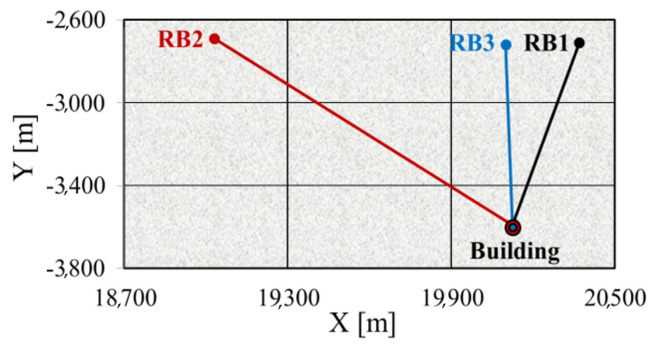
Locations of the rock bursts foci and the building.

To analyse the numerically determined dynamic responses of low-rise buildings, three representative rock bursts were selected as the excitations of building vibrations. The mine-induced tremors, denoted as RB1, RB2, and RB3, were singled out from hundreds of recorded rock bursts. The three tremors vary in terms of parameters: energy level, epicentral distance, vibration duration, peak horizontal velocity, main frequency intervals, and direction of wave arrival. Energy levels, epicentral distances, vibration durations, peak horizontal velocities and main frequency intervals of the considered shocks are given in Table 2, whereas the wave propagation directions are shown in a simplified manner in Figure 3. Locations of the rock bursts foci and the building in the local seismic coordinate system (X, Y) are also presented in this figure.

The observed differences in excitation characteristics significantly influence the subsequent structural modal responses. RB1 excitation is characterised by the highest vibration amplitudes (maximum velocities up to 0.031 m/s in the x-direction and 0.040 m/s in the y-direction) and relatively low dominant frequencies (4.40–8.55 Hz), which promotes stronger activation of low-frequency structural modes. RB2 excitation exhibits considerably lower amplitudes (0.009–0.010 m/s) and higher dominant frequencies (10.74–15.14 Hz), resulting in a reduced overall response magnitude while preferentially exciting intermediate-frequency modes. RB3 excitation has the lowest amplitudes (0.005–0.007 m/s) but the highest dominant frequencies (13.43–21.24 Hz). Consequently, its response is expected to be dominated by higher-order vibration modes, while the lower input energy limits the overall response amplitudes.

Furthermore, the differences in vibration duration may affect the persistence of modal oscillations. RB1 shows the longest duration in the x-component (1.61 s), whereas RB3 exhibits the longest duration in the y-component (1.57 s), suggesting a greater potential for sustained modal participation in the corresponding directions. Overall, the progression from RB1 to RB3 corresponds to a shift from high-amplitude, low-frequency excitation toward lower-amplitude, higher-frequency excitation, leading to a transition from predominantly lower-mode structural responses to responses involving increasingly higher vibration modes.

The focus of this study was on horizontal mine-induced vibrations because of their dominant effect on buildings. In typical low-rise buildings situated in mining areas, the vertical inertial forces generated by the vertical component of ground vibrations are generally lower than the corresponding static gravitational loads. Consequently, the influence of vertical ground-motion components was considered negligible and was therefore not included in the analyses presented in this article. The identified horizontal and vertical vibration mechanisms are applicable to low-rise buildings with up to five storeys, storey heights of no more than 3 m, and a mixed transverse–longitudinal bearing wall system. Therefore, the conclusions drawn in this study should be interpreted within the limits of these structural and geometric characteristics. The recommendations and limitations were confirmed by the results of in situ tests in the case of mining vibrations [4,23].

**Figure 4:**
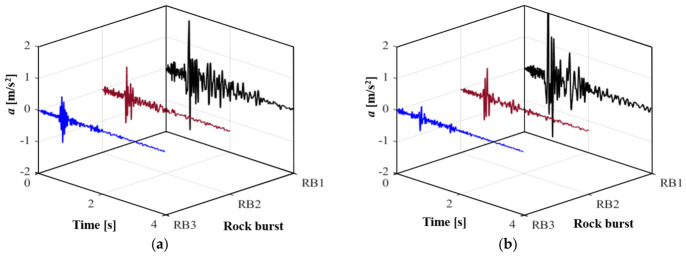
In situ recorded free-field time history vibrations in the transverse x (**a**) and longitudinal y (**b**) directions for the RB1, RB2, and RB3 mine-induced tremors.

In situ recorded free-field time history horizontal accelerations (a) in the transverse (x) and longitudinal (y) directions for the RB1, RB2, and RB3 mine-induced tremors are shown in Figure 4. Naturally, differences in mining tremor parameters result in differences in the recorded vibrations next to the building.

**Figure 5:**
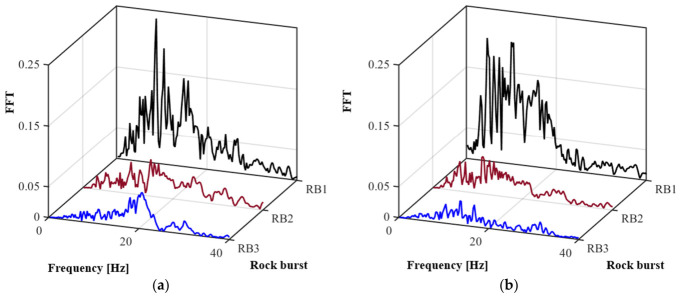
Fourier spectra (FFT) corresponding to in situ recorded free-field time history vibrations in the transverse x (**a**) and longitudinal y (**b**) directions for the RB1, RB2, and RB3 mine-induced tremors.

Additionally, Fourier spectra (FFT) corresponding to in situ measured free-field time history accelerations are presented in Figure 5. Differences in Fourier spectra for the RB1, RB2, and RB3 shocks are also visible.

Experimental data containing recorded ground vibration time histories next to the building were used as vibration excitations in further numerical analysis.

## 3. 3D FEM Numerical Model

The construction of a numerical model of the building and dynamic analyses were carried out using the finite element method software ANSYS Mechanical 18.2 [24]. The soil–structure interaction was taken into account. ANSYS was selected due to its advanced capabilities in dynamic analysis, efficient modal analysis tools, and high flexibility in defining material parameters and boundary conditions. The use of parametric scripting allowed for fast modification of material properties of load-bearing walls and reduced the risk of modelling errors. In addition, the possibility of including damping in both the structure and the soil model is crucial for realistic simulation of building response to mining-induced vibrations.

The numerical model of the structure was developed on the basis of detailed architectural documentation, including ground and first-floor plans and vertical cross-sections as well as cross-sections of all elements of the building. The finite element model includes all main structural components with appropriate material parameters and element types assigned.

**Figure 6:**
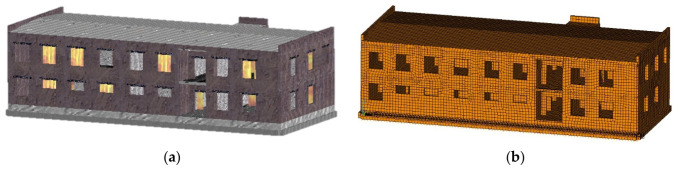
3D FEM building model: (**a**) model geometry; (**b**) finite element mesh.

The three-dimensional finite element model of the analysed building, together with the applied finite element mesh, is presented in Figure 6. The figure illustrates both the geometric representation of the structure and its discretisation used in the numerical analyses.

The numerical model of the building was created as a full three-dimensional structure, including all main structural parts from the foundation to the roof. The model includes foundation footings and tie beams, foundation walls, ground-floor slab and structural system, first-floor slab and structural system, knee walls, and the roof structure. Each structural layer was introduced with its actual geometry, thickness, and material parameters reflecting real construction conditions.

The adopted finite element discretisation was previously verified by validation of the numerical model against in situ measurements performed on the analysed building [4]. The finite element mesh was generated using the standard automatic meshing procedure available in ANSYS Mechanical APDL [24]. This procedure includes automatic element shape checking and mesh quality control, ensuring appropriate element quality for structural dynamic analyses. Good agreement between the numerical and experimental results was obtained. This confirmed that the modelling approach provides sufficient accuracy for the purposes of the present calculations.

The same finite element mesh was applied in all analysed building variants. Therefore, the discretisation remained identical for all numerical models, and the influence of the adopted mesh on the calculated dynamic responses remained the same in all analysed cases. The mesh was kept unchanged throughout all analyses. Therefore, the adopted finite element discretisation was considered appropriate for the purposes of the present study.

To build the numerical model, two types of finite elements available in the ANSYS system were applied: the four-node SHELL181 element and the two-node BEAM188 element [24]. The SHELL181 elements, with six degrees of freedom at each node, were used to model all surface structural components, including load-bearing walls, partition walls, floor slabs, foundation walls, stairwell, and roof panels. Linear beam-type elements BEAM188, also with six degrees of freedom per node, were applied to model linear structural members such as continuous strip foundations, foundation tie beams, and ceiling ties.

The foundation footings were modelled using BEAM188 elements with cross-sections of 0.4 m × 0.7 m and 0.4 m × 0.5 m. To increase the stiffness of the foundation system under mining-induced vibrations, reinforced-concrete foundation tie beams with square cross-sections of 0.3 m × 0.3 m were introduced. Above the footings, the foundation walls with a thickness of 0.25 m were modelled as reinforced-concrete shell elements. For these elements, typical reinforced-concrete material parameters were assigned.

On the ground floor and the first floor, load-bearing walls were modelled using SHELL181 elements. In order to investigate the influence of wall material on the dynamic response of the building, several variants of wall materials were considered, including reinforced concrete, brick, cellular concrete, and sand–lime bricks with different strength classes. In all numerical variants, the partition walls were assumed to be made of brick and were modelled with a constant thickness of 0.12 m using SHELL181 elements.

The floor structures were modelled as reinforced-concrete slabs using SHELL181 elements. Based on the construction documentation, the thickness of the first-floor slab was assumed to be 0.132 m, while the ground-floor slab thickness was 0.20 m. The slabs are supported by load-bearing walls and reinforced-concrete ceiling ties. The ceiling ties, modelled using BEAM188 elements with cross-sections of 0.24 m × 0.24 m, ensured proper load transfer and spatial stiffness of the structure. The reinforced concrete of the ties was described by an elastic modulus of 27.0 GPa and a density of 2500 kg/m^3^.

The reinforced-concrete stairwell was also modelled using SHELL181 elements, with the stair slab thickness equal to 0.10 m. The roof structure was defined as prefabricated reinforced-concrete troughed panels supported on brick knee walls.

**Figure 7:**
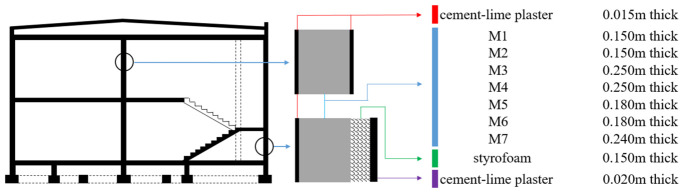
Layers in external and internal load-bearing walls.

For the modelling, seven materials were considered for load-bearing walls. Each external wall includes a load-bearing layer, a thermal insulation layer (styrofoam), and two cement–lime plaster layers. Each internal load-bearing wall consists of only three layers: a load-bearing layer and two cement–lime plaster layers. Figure 7 shows the layout of wall layers, the applied materials, and their thicknesses.

The load-bearing walls are modelled using SHELL181 elements, which allow several material layers to be defined in one element and enable separate stress calculation for each wall layer.

A linear-elastic material behaviour was assumed for all structural elements of the building. It is based on long-term inspections of the real structure, which did not reveal any structural damage in the load-bearing walls, lintels, floors, or foundations. The building with brick load-bearing walls had been exposed for many years to dynamic loads caused by mining tremors of different intensities. Only small architectural damage, such as minor cracks in plaster and paint layers, was observed. This means that stresses and strains remained within the elastic range of the materials.

The flexibility and damping of the subsoil were included in the numerical model by using spring–damper elements COMBIN14 in the horizontal (x and y) and vertical (z) directions. These elements allow the soil to deform elastically and dissipate energy under dynamic loads from mining tremors [24]. The stiffness of the springs was calculated using the Savinov soil model and the soil reaction coefficient Cz [25]. Based on in situ measurements and typical geotechnical conditions for the analysed area, a dynamic soil parameter Cz = 50 MPa was assumed.

To define the damping of the soil, a linear viscous damping model was applied in the COMBIN14 elements. The damping coefficient c of the COMBIN14 dashpot elements was determined according to the Lysmer analogue [26,27,28,29]: c = ρVsA, where ρ is the soil density, Vs is the shear wave velocity and A is the foundation–soil contact area. The damping coefficients were determined using typical values for medium-dense sands: soil density of 1800 kg/m^3^ and shear wave velocity of 200 m/s. The total contact area of the foundation was also taken into account. These parameters are consistent with soil category B according to Eurocode 8 [30], which corresponds to the geotechnical conditions at the building site in the Upper Silesian Coalfield area. For ρ = 1800 kg/m^3^ and Vs = 200 m/s, the unit damping coefficient equals 3.6 × 10^5^ Ns/m, which was multiplied by the corresponding contact area assigned to each spring–damper element.

Several methods have been proposed in the literature for the identification of modal damping ratios. One such method is the transfer-function-based method proposed in [31]. The application of this method requires acceleration measurements at both the foundation and roof levels to determine the roof-to-basement transfer function. Since roof acceleration measurements were not available in the present study, a damping ratio of 5% of critical damping was assumed in accordance with the recommendations of Eurocode 8 [30] for conventional building structures.

The issue of soil–structure interaction (SSI) was not considered in the present study in detail. However, in the context of mining-induced vibrations, SSI effects have been extensively investigated in our previous research, as reported in References [4,32]. The results of those studies demonstrated that accounting for soil flexibility leads to a reduction in the natural frequencies of the analysed building compared with the fixed-base model. This phenomenon is attributed to the additional compliance introduced by the deformable foundation, which increases the overall flexibility of the soil–structure system. Consequently, the inclusion of SSI provides a more realistic representation of the dynamic behaviour of buildings subjected to mining-induced ground motions and enables a more accurate assessment of their vibration response.

Dynamic analyses were performed using ANSYS Mechanical software. Structural response was calculated for three measured kinematic excitations representing typical mining tremors recorded in the region. The time integration was carried out using the Newmark algorithm and Rayleigh damping [33], with a 5% damping ratio applied. Two orthogonal horizontal components of acceleration were introduced simultaneously using spring–damping elements at foundation nodes.

The 3D FEM model was validated using experimental data from in situ measurements carried out on a real building with load-bearing walls made of brick (M4). Good agreement between numerical and experimental data was obtained, which confirmed the correctness of the adopted modelling approach [4].

## 4. Results and Discussion of Building Dynamic Responses

### 4.1. Introductory Remarks

**Figure 8:**
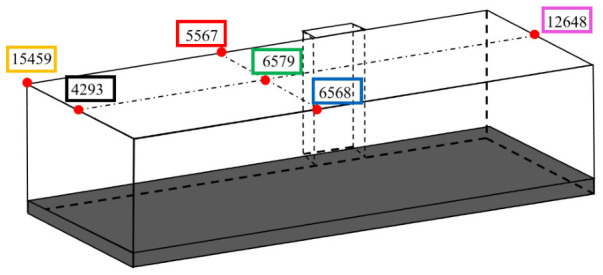
Position and numbers of the selected FEM nodes.

To analyse the impact of the choice of the place of numerical estimation on the calculated building dynamic mine-induced response, six points (FEM nodes) were identified in each low-rise building variant (M1 to M7). The selected nodes are located at the ceiling level of the second floor of the building, at the places of connections of the transverse and longitudinal load-bearing walls. The detailed position of the chosen FEM nodes and their numbers are shown in Figure 8.

The dynamic responses numerically obtained in the selected nodes were compared. Such comparisons were performed for each variant of low-rise building (M1 to M7) in the three cases of vibration excitations by rock bursts RB1, RB2, and RB3. Both dynamic responses in the form of displacements and accelerations were analysed. For both displacements and accelerations, time histories in directions parallel to the building axis, peak values of vibration components, resultant vibrations, and Fourier spectra were taken into account. Results and discussion for comparisons of low-rise building dynamic responses, in the form of displacements and accelerations, are presented in Section 4.2 and Section 4.3, respectively.

### 4.2. Displacements

**Figure 9:**
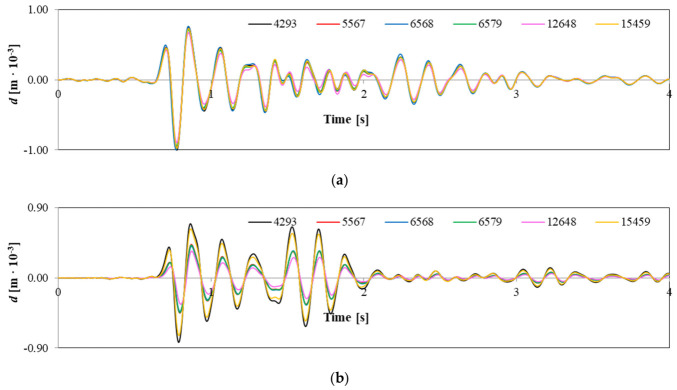
Computed responses at selected nodes for the RB1 mining-induced tremor for material M1 in the x (**a**) and y (**b**) directions.

**Figure 10:**
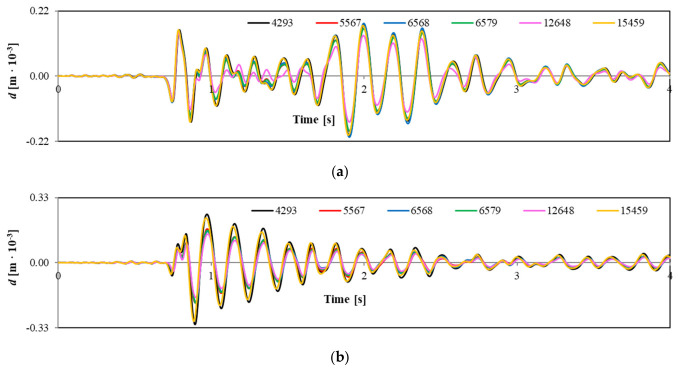
Computed responses at selected nodes for the RB2 mining-induced tremor for material M3 in the x (**a**) and y (**b**) directions.

**Figure 11:**
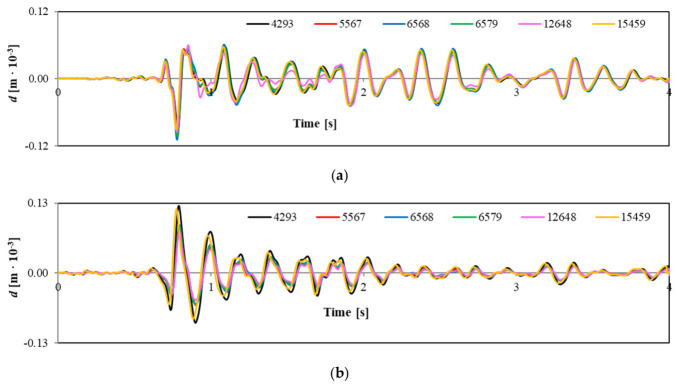
Computed responses at selected nodes for the RB3 mining-induced tremor for material M4 in the x (**a**) and y (**b**) directions.

Exemplary plots of time history displacement responses in x and y-directions for the selected nodes are shown in Figure 9, Figure 10 and Figure 11. Figure 9 presents the low-rise building dynamic response for the RB1 mining-induced tremor for material M1, Figure 10 shows the case of material M3 and rock burst RB2, whereas Figure 11 concerns the rock burst RB3 and material M4.

The presented vibration response examples (see Figure 9, Figure 10 and Figure 11) of the building determined for the same rock burst and the same material at different points of the second floor of the low-rise building show significant differences. These differences mainly concern the values of displacements at given moments in time and are much greater for the longitudinal y-direction. Some slight phase shifts can also be observed in the response time histories calculated for different nodes, especially for the low-intensity excitation RB3. Analysis of such drawings performed for all considered cases (3 shocks × 7 building material variants × 2 vibration directions) leads to similar conclusions.

The computed maximum displacement values in the x- and y-directions at the second-storey level of the building for selected nodes, considering the analysed structural materials and representative mining-induced tremors, are presented in Table 3.

**Figure 12:**
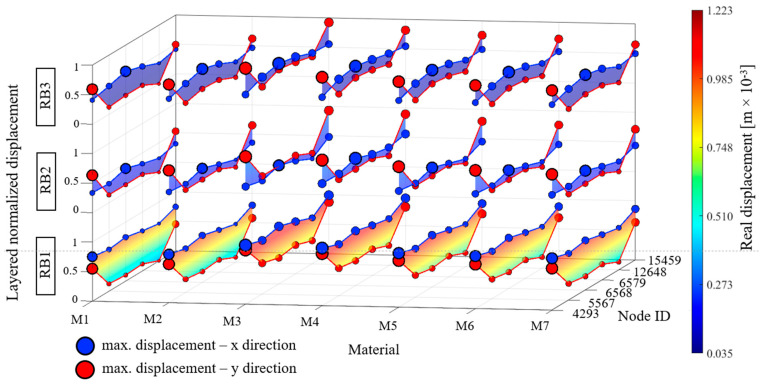
Normalised peak values of displacements computed in chosen nodes, seven materials, and rock bursts RB1, RB2, and RB3.

Additionally, using data from Table 3, the normalised peak displacement values at individual nodes in the transverse (x) and longitudinal (y) directions, considering the three analysed mining tremors and seven structural materials, are presented in Figure 12. All displacements were normalised to the range [0, 1] to enable direct comparison of graphs for mining tremors of different intensities. For this purpose, separately in the case of each rock burst, the following formula was used: (d − d_min_)/(d_max_ − d_min_), where d—node displacement value, d_min_—minimum displacement value, and d_max_—maximum displacement value. Additionally, information about the value of the displacement is reinforced by the size of the markers: larger dots correspond to larger displacements, and nodes with the maximum value in a given series are marked with a black outline. In turn, the actual displacement values can be read from the colour maps—the scale is placed next to the figure.

For quantitative displacement data analysis support, the relative response amplification coefficients of materials M2–M7 compared to reinforced concrete M1 were determined. The mean values (MV) of the displacement responses in the considered nodes were taken into account for this purpose according to the relation Mi/M1, i = 2 − 7. The obtained results are presented in Table 4.

From Table 3, it follows that under the highest excitation level (RB1), displacement amplitudes in every considered node reach their maximum values. The maximum values appear in node No. 4293 and depend on the level of excitation, the material and the direction of the response. In the case of excitation RB1, for each analysed node and material (M1–M7), the displacements in the transverse x-direction are greater than those in the longitudinal y-direction and remain in x and y-directions within the ranges 1.005 (M1)–1.223 (M3) mm and 0.827 (M1)–1.121 (M4) mm, respectively. Rock burst RB2 generated a weaker horizontal response on the second floor, which can be attributed to its lower energy compared to rock burst RB1. In the transverse (x) direction, the largest displacements were observed at node No. 6568, located at the midpoint of the external longitudinal wall of the building, with values ranging from 0.183 mm (M1) to 0.234 mm (M4). In contrast, in the longitudinal (y) direction, the maximum displacements were greater than those in the transverse direction. The peak displacements along the y-axis occurred at node No. 4293 and ranged from 0.233 mm (M1) to 0.314 mm (M3). For the weakest rock burst RB3, the maximal displacement was achieved for every considered node and material. The maximal values are in node No. 4293, remaining in the range 0.092–0.138 mm. Otherwise, in the y-direction, the maximal displacements are greater, appear in node No. 4293 and remain in the range 0.096 (M1)–0.138 (M3) mm. The analysis of the data summarised in Table 3 indicates that RB1 induces the highest level of structural excitation, RB3 exhibits a distinctly low-energy seismic profile, whereas RB2 represents an intermediate ground motion characterised by noticeably greater response along the y-axis (e.g., M1: y = 0.233 mm vs. x = 0.167 mm).

In the longitudinal direction (y), the highest displacement values occur at node 4293 for all considered material and rock burst variants, which is clearly visible in Figure 12 and also in Table 3. The peak displacement values computed for the other considered nodes could definitely be smaller. For example, in the case of material M1, these peak values computed at node 12648 constitute only 42%, 40%, and 36% of the values determined for node 4293 for excitations RB1, RB2, and RB3, respectively. In almost all variants of materials and shocks (81% of all cases), the displacement values at node 12648 do not exceed 50% of the value at node 4293. These differences are smaller only in four cases (shock RB3, materials M3–M6).

In contrast, in the transverse (x) direction, the node with the maximum value of displacement depends on the intensity of vibration excitation. For all material variants, it is node 4293 in the case of rock burst RB1, but it is node 6568 in the case of lower energy mining tremors RB2 and RB3 (see Figure 12 and Table 3). The differences in the peak displacement values determined at various nodes are smaller than in the case of the longitudinal direction (y). For all considered materials and rock bursts, the ratio of the smallest calculated peak displacement value to the largest one is in the range of 75–89%.

**Figure 13:**
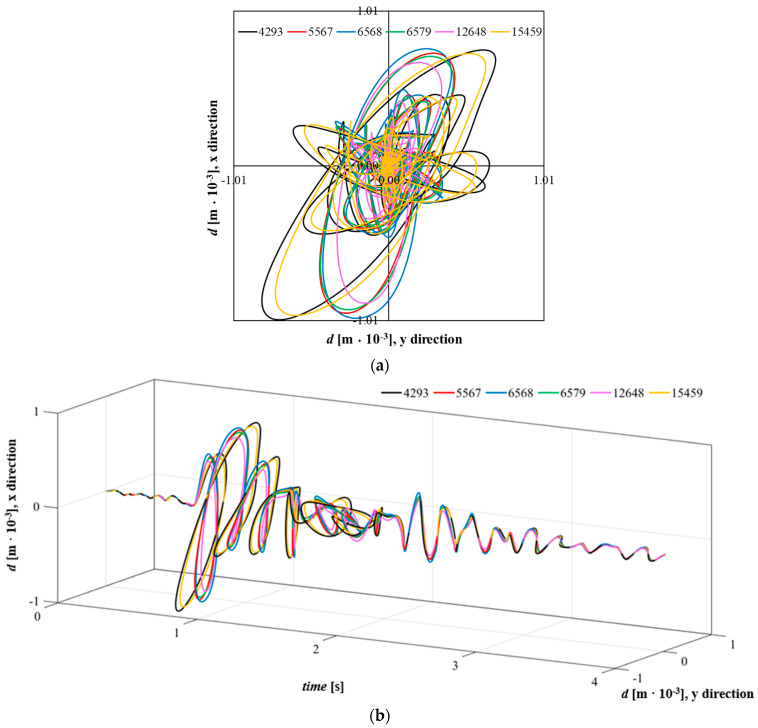
Comparison of resultant displacement 2D trajectories (**a**) and 3D trajectories as a function of time for the analysed nodes (**b**)—illustrated for mining tremor RB1 and material M1.

The resultant displacements vs. time can also be visualised using 3D plots, which provide an illustrative representation of how these displacements evolve at six selected nodes of the second floor. For instance, Figure 13 depicts the resultant displacement responses for load scenario RB1 and material configuration M1 across all analysed nodes.

As an example, the interpretation of the resultant in-plane displacements vs. time and scenario RB1, across all six nodes and materials M1–M7, is presented below. All values are reported in millimetres (mm), and the time of occurrence (in seconds) is given for each maximum. Table 5 condenses the maximum resultant displacement for each node × material pair, with its time of occurrence. Values are rounded to 0.001 mm and 0.001 s for clarity.

After reviewing all computed data for scenario RB1, the global maximum occurs at node 15459/Material M3: 1.543 mm at t = 0.780 s. For M4–M7, node 15459 also governs, with maxima between 1.370 and 1.541 mm at t ≈ 0.782–0.786 s. For material M1, the largest response is at node 4293 (1.259 mm in 0.782 s), while node 15459 remains second (1.177 mm in 0.782 s). Node 6579 is an order of magnitude less responsive (≤0.133 mm across all materials), suggesting proximity to constraints or a locally stiff/stabilised region.

The peak times cluster is tightly around 0.772–0.786 s across all nodes and materials, which indicates temporal coherence. Such temporal alignment usually indicates a global dynamic phenomenon (e.g., the dominance of a principal vibration mode or a synchronised transient) under the RB1 excitation. While the excitation profile is not included, the coincidence of peaks supports a system-wide response rather than purely local effects. In view of the spatial concentration of response demand, the most pronounced envelopes are observed at node 15459, which governs six out of seven material configurations. Nodes 5567 and 6568 consistently exhibit elevated, albeit slightly lower, peak magnitudes, ranging approximately between 1.14 mm and 1.26 mm. By contrast, node 6579 remains low in all materials (≈0.09–0.13 mm). This spatial distribution suggests gradients in flexibility or boundary constraint influence within the model. Considering material sensitivity on dynamic response, it follows that although all materials have the same temporal pattern, the magnitude ordering varies—M3–M4–M5–M6–M7 tend to produce the largest displacements at the critical node (15459), while M1 peaks at 4293. This may reflect material property contrasts (e.g., stiffness/density) and their modal participation under the RB1 scenario. The observed spatial concentration of peak response magnitudes indicates pronounced heterogeneities within the structural system, likely driven by stiffness gradients and boundary constraint effects in the numerical model. Localisation of the largest envelopes at node 15459—governing six out of seven material configurations—combined with consistently elevated yet slightly smaller peaks at nodes 5567 and 6568, suggests that demand is controlled by non-uniform flexibility rather than a uniformly distributed deformation field. This pattern aligns with scenarios where modal participation and load-path continuity amplify local displacements while attenuating response in peripheral regions. From a design and assessment perspective, these findings underscore the need to refine spatial distributions of material properties and boundary conditions, prioritise instrumentation at governing nodes, and consider targeted mitigation strategies such as localised stiffness augmentation to reduce peak demands without imposing excessive global stiffening. Based on the analysis of all computed resultant displacements for RB1, four diagnostic and design-relevant points are recommended: (i) node 15459 as the primary governing location (maxima for M2–M7 and global maximum for M3), (ii) nodes 5567 and 6568 as secondary high-response points for redundant monitoring and cross-validation, (iii) node 4293 as a material-specific hotspot governing M1, and (iv) node 6579 as a baseline reference with minimal amplitudes for normalisation and boundary-effect assessment. This set provides a balanced basis for model validation, response-reduction strategies, and sensor placement planning under RB1.

**Figure 14:**
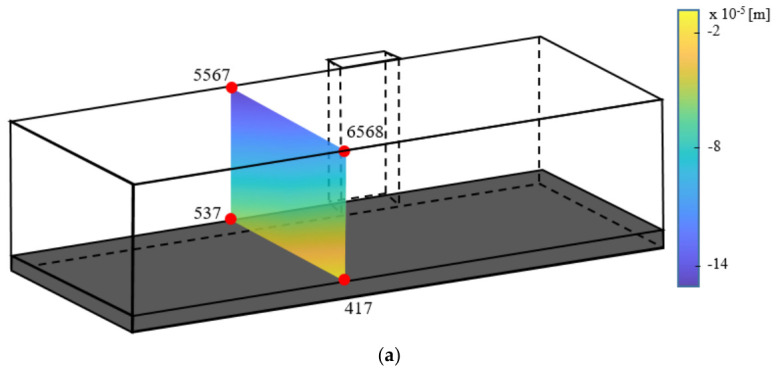

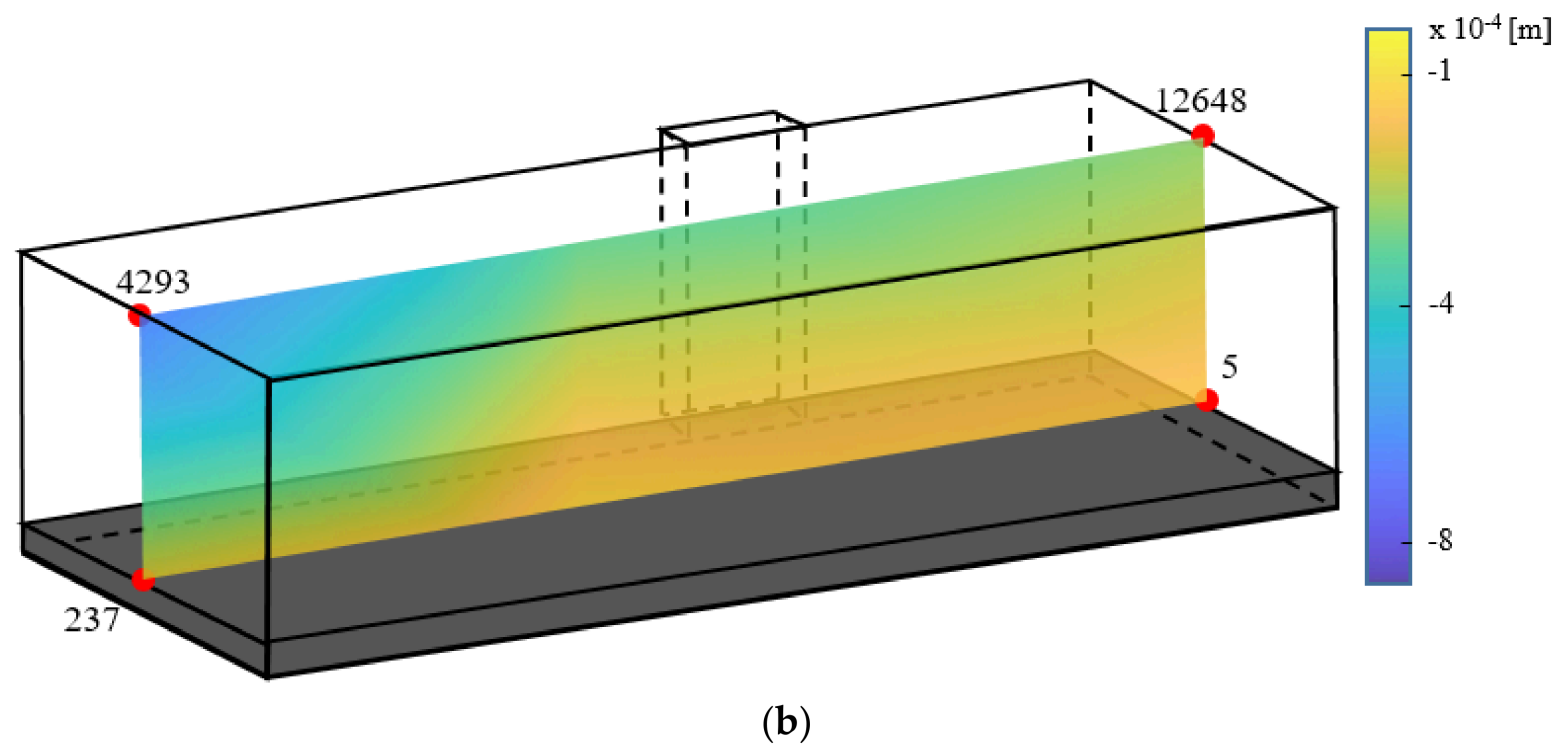
Displacement maps of the responses of two selected walls (material M1) on the RB1 rock burst computed for the 0.79 s in the transverse x-direction (**a**) and in the longitudinal y-direction (**b**), respectively.

Significant differences that may appear in the displacement responses of building various points to mining tremor excitations at the same time are also confirmed by the displacement maps determined for the building’s load-bearing walls. Figure 14 illustrates this effect using an example.

Table 6 summarises the dominant frequencies identified in the dynamic response of the structural model at selected nodes located at the second-floor level. The tabular data reflect the influence of the analysed material variants (M1–M7) and mining-induced tremors RB1, RB2, and RB3.

**Figure 15:**
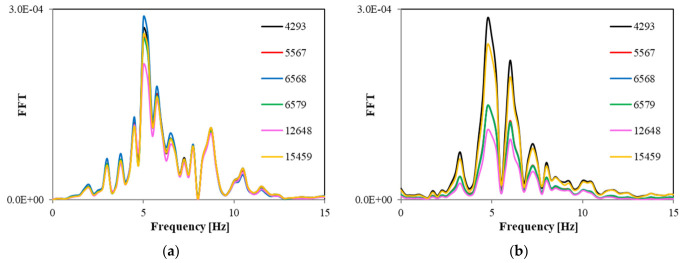
FFT corresponding to response in x-direction (**a**) and y-direction (**b**) for material M1 for the RB1 mine-induced tremor based on calculated responses shown in Figure 9.

The analysis of dominant frequency values in response to the most intensive tremor RB1 reveals that the response in the x-direction exhibits high stability, with most nodes and material configurations converging to approximately 5 Hz. This consistency suggests that the structural stiffness and mass distribution in this direction govern the dynamic characteristics, regardless of material variability. The minimum observed frequency, 4.5 Hz, occurs at node No. 12648 for material M4, indicating a localised reduction in stiffness or mass concentration. Conversely, the maximum frequency, 5.25 Hz, is associated with material M3 and appears at nodes No. 4293 and 15459, which may correspond to regions of increased rigidity. In the y-direction, the dominant frequency remains constant at 4.75 Hz across all nodes and materials. This value closely matches one of the principal components of the excitation spectrum in this direction (see Figure 5b), confirming that the response is predominantly modal rather than forced. Such behaviour indicates that the structural system resonates with the excitation in the y-direction, amplifying the modal contribution. In contrast, the transverse x-direction exhibits a purely forced response, as the dominant excitation frequency in this direction is significantly higher—8.8 Hz (see Figure 5a)—than the frequencies of the system response. This discrepancy implies that the mining tremor does not activate the structural modes in the x-direction, and the observed vibrations are governed by the characteristics of the external forcing rather than the inherent dynamic properties of the structure. For example, the computed FFT plots for x and y-directions and material M1 for the selected nodes are presented in Figure 15.

For the RB2 tremor, analysis of dominant frequency values in the x-direction response indicates that the frequencies remain within the range of 5–5.75 Hz for materials M1–M7. This range is substantially lower than the dominant frequencies present in the x component of the excitation, which confirms that the response does not exhibit a modal character but is primarily forced. Furthermore, the band of dominant frequencies in the x-direction response is slightly narrower compared to the y-direction, where the range extends from 5 to 6 Hz for materials M1–M7. Although the excitation in the horizontal y-component covers a broad frequency spectrum, the lowest significant frequency of 6.1 Hz can be distinguished. This observation provides a basis for concluding that, in the y-direction, the dynamic response of the selected nodes in the building model is predominantly modal.

The dominant frequency values in response to tremor RB3 strongly depend on the frequency characteristics of the forcing. The computed dominant frequency values in x and y-directions (5.25 and 5.0 Hz, respectively) and in the node responses are relatively low and significantly smaller than the dominant frequencies present in the x and y components of the excitation (comp. Figure 5). This observation suggests that, for tremor RB3, the structural response at the selected nodes is governed primarily by the system’s natural modes rather than by the characteristics of the external forcing. In other words, the excitation does not strongly activate high-frequency components, allowing the modal properties of the structure—such as stiffness and mass distribution—to dominate the dynamic behaviour. This modal predominance is typical when the forcing frequencies lie outside the range of the structure’s natural frequencies, resulting in resonance effects being minimal and the response reflecting intrinsic dynamic characteristics.

These findings highlight the directional dependency of dynamic behaviour under mining-induced excitations and underscore the importance of considering both modal and forced components when assessing structural performance under transient loads.

However, the data from Table 6, as well as the FFT curves (presented example graphs from Figure 15 and those for the other material variants and rock bursts), indicate a very small influence of the node location on the determined dominant frequency of vibration displacements in the time domain. The calculated dominant frequency for vibration displacements is practically independent of the node selection.

### 4.3. Accelerations

**Figure 16:**
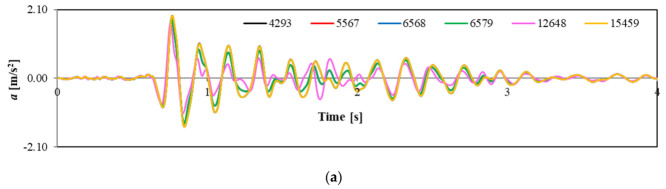

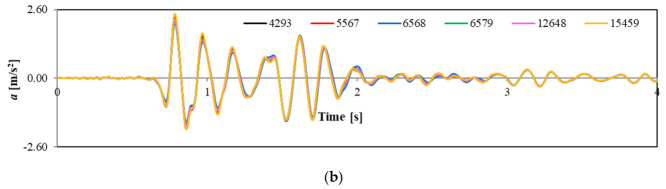
Computed acceleration responses at selected nodes for material M1 in the x (**a**) and y (**b**) directions under the RB1 excitation.

**Figure 17:**
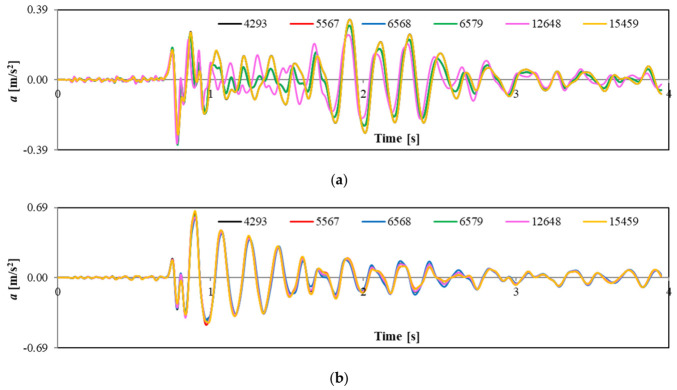
Computed acceleration responses at selected nodes for material M6 in the x (**a**) and y (**b**) directions under the RB2 excitation.

**Figure 18:**
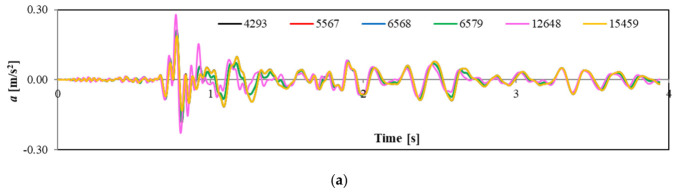

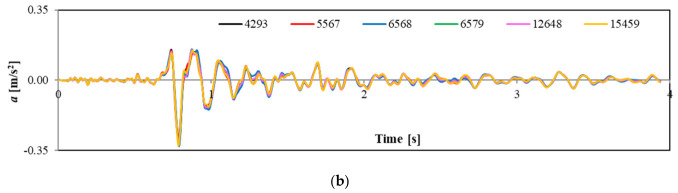
Computed acceleration responses at selected nodes for material M2 in the x (**a**) and y (**b**) directions under the RB3 excitation.

Exemplary plots of time history acceleration responses in x and y-directions for the selected nodes are shown in Figure 16, Figure 17 and Figure 18. Figure 16 presents the low-rise building dynamic response for the RB1 mining-induced tremor for material M1, Figure 17 shows the case of material M6 and rock burst RB2, whereas Figure 18 concerns the rock burst RB3 and material M2.

Similarly to the case of displacement time history responses (see Section 4.2), the presented examples (see Figure 16, Figure 17 and Figure 18) and the rest of the considered samples of acceleration responses of the building and rock burst variants, determined for the same rock burst and the same material at different points of the second floor of the low-rise building, show significant differences. However, in contrast to the displacement responses, larger differences for accelerations are visible for the transverse x-direction. These differences concern not only the values of accelerations at given moments in time but also significant phase shifts in vibration curves for the considered nodes. In the longitudinal y-direction, the differences in the accelerations in time domain calculated at the six selected nodes are relatively small.

The computed maximum acceleration values in the x- and y-directions at the second-storey level of the building for selected nodes, considering the analysed structural materials and representative mining-induced tremors, are shown in Table 7.

**Figure 19:**
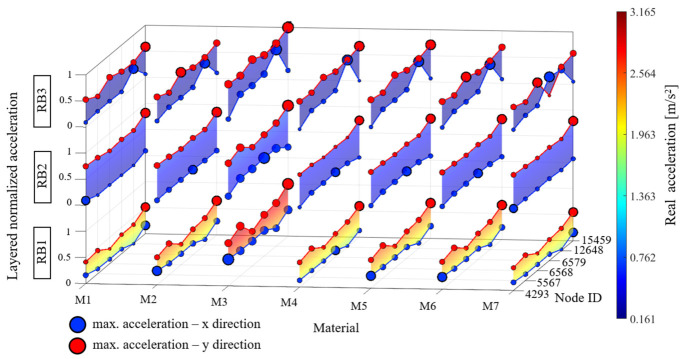
Normalised peak values of accelerations computed in chosen nodes, seven materials, and rock bursts RB1, RB2, and RB3.

For a better visualisation of the results from Table 7, the peak acceleration values were normalised to the range [0, 1]. A similar procedure was used as in the case of displacements (see Section 4.2). A graphical presentation, which uses analogous notations as in the case of displacements, is shown in Figure 19.

For a better quantitative comparison analysis, the coefficients of variation (CV) and the mean values (MV) of the responses of the seven materials in the considered nodes were also calculated. The values of CV and MV are shown in Table 8.

Additionally, the amplification coefficients of acceleration for the M2 to M7 materials compared to reinforced-concrete M1 were determined in a similar way as in the case of displacements (see Section 4.2). As with the displacements, the mean values of the accelerations over six nodes were taken into account. The obtained results are presented in Table 9.

Under RB1 rock burst excitation, the highest dynamic response accelerations were observed for material M3, reaching 2.432 m/s^2^ in the x-direction (node No. 4293) and 3.165 m/s^2^ in the y-direction (node No. 15459). In contrast, for RB2 and RB3, the computed nodal accelerations were substantially lower, reflecting the reduced intensity of these events. For RB2, a marked decrease in peak acceleration values was noted in both directions, with maxima of 0.465 m/s^2^ at node No. 12648 (x-direction) and 0.744 m/s^2^ at node No. 15459 (y-direction) for material M3. For RB3, the maximum accelerations were 0.349 m/s^2^ (x-direction) and 0.406 m/s^2^ (y-direction). Comparison of these results indicates that material M3 consistently exhibits the highest acceleration values across all scenarios, suggesting its lower capacity for vibration damping and, consequently, greater susceptibility to dynamic loading—comp. Table 6. Analysis of the data presented in Table 6 indicates that material selection has a significant influence on peak acceleration under rock burst conditions. The position of the node had only a minor effect, suggesting that global dynamic properties dominate over local variations. Materials with lower damping capacity (e.g., M3) exhibit higher dynamic responses, which should be taken into consideration in structural design.

Comparison of Figure 19 with Figure 12 and Table 6 with Table 3 leads to the conclusion that the differences in the peak acceleration values determined at different nodes are smaller than for the displacements in analogous cases of materials and shocks. However, some differences can also be observed in the peak acceleration values calculated at individual nodes, especially in the transverse (x) direction for the low-intensity RB3 shock. For example, in the case of materials M3, M4, and M5, these peak values computed at node 15459 constitute only 58%, 53%, and 58% of the values determined for node 12648, respectively.

A quantitative comparison of the resultant peak accelerations indicates that material M3 exhibits the highest mean response (2.93 m/s^2^ in the case of RB1 rock burst), significantly exceeding the values obtained for the remaining materials for all of the considered rock bursts. The coefficients of variation range from 0.8% to 22.6%, demonstrating varied dispersion of responses among the analysed nodes. M1 shows the most uniform behaviour (CV < 12.2%), whereas M4 exhibits the greatest variability (CV = 22.6%). These results confirm that M3 is the most dynamically sensitive material, while M1, M4, and M7 provide the lowest and most stable dynamic responses.

**Figure 20:**
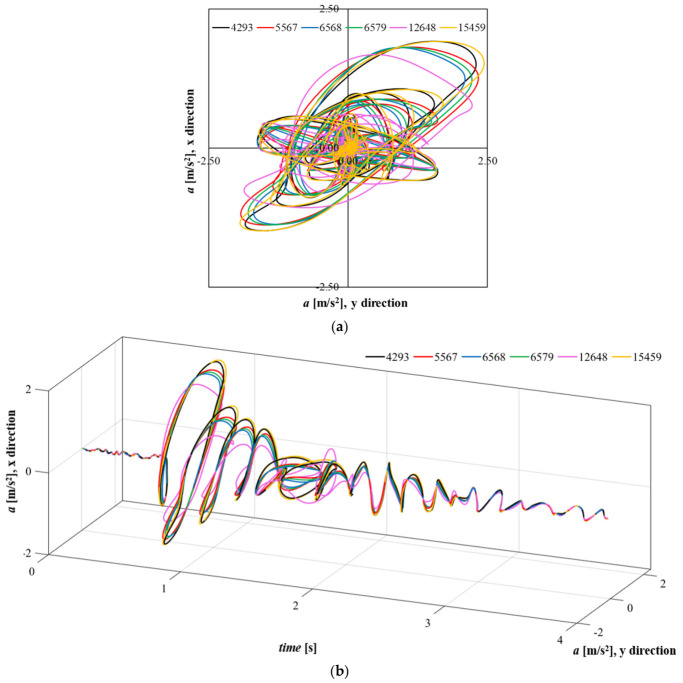
Comparison of resultant acceleration 2D trajectories (**a**) and 3D trajectories as a function of time for the analysed nodes (**b**)—illustrated for mining tremor RB1 and material M1.

The resultant acceleration vs. time can also be visualised using 3D plots, which provide an illustrative representation of how these accelerations evolve at six selected nodes of the second floor. Using all the data concerning computed acceleration in the x and y-directions, the resultant (vector) in-plane accelerations at the selected nodes and materials M1–M7 were calculated, along with the resultant acceleration and time of occurrence. Similarly to the resultant displacements, the resultant accelerations were calculated. For instance, Figure 20 depicts the resultant acceleration responses for load scenario RB1 and material configuration M1 across all analysed nodes.

To characterise node representativeness, the values of maximum resultant computed acceleration across M1–M7 and the time of occurrence were computed. All computed peak resultant accelerations and occurrence times (M1–M7, all nodes) are presented in Table 10. The largest mean peak and tight timing occur at node No. 15459. Nodes No. 4293 and 5567 exhibit similarly coherent timing with lower amplitudes. For each material (across all nodes), the largest resultant acceleration in the entire model occurs at node 15459—comp. Table 10. The model exhibits a dominant, coherent peak of the relevant acceleration across all materials, with amplitudes ranging from ≈2.94 to ≈3.60 m/s^2^ and synchronous timing concentrated in a narrow window (≈0.778–0.784 s). This temporal coherence is characteristic of a single, short-duration excitation with a shock-type input RB1.

When the peak times are aggregated across materials at each node, the dispersion is small and similarly low at nodes 4293 and 5567. This indicates that the entire structure responds nearly simultaneously, with node 15459 showing both the largest amplitudes and very tight timing coherence. A practical representative should (i) capture high amplitudes across materials and (ii) exhibit stable, coherent peak timing. Ranking nodes by the means of per-material peak magnitudes and penalising large timing dispersion identifies node No. 15459—the highest mean peak amplitude across M1–M7, and low timing dispersion, node No. 4293—slightly lower mean amplitude, and similarly coherent timing and node No. 5567—coherent timing, but further reduced amplitudes. Analysis of the results indicates that node No. 15459 is the most representative location for evaluating the influence of the RB1 shock on the model’s dynamic response. It concentrates the global maxima for all materials and shows a well-aligned peak time, providing a conservative and robust vantage point for subsequent analyses. Among the materials examined, M3 consistently demonstrates the highest peak acceleration (approximately 3.60 m/s^2^ at 0.780 s), which may indicate reduced stiffness, increased local mass participation, or a closer spatial relationship to the RB1 input within its material domain.

A summary of the dominant frequencies of the building’s dynamic acceleration responses at the specified nodes and materials is provided in Table 11.

Under RB1 excitation, the dominant response frequencies in the x-direction for the analysed nodes remain stable within a narrow range from 5.0 Hz (M1, M2, M4, M5, M6, and M7) to 5.25 Hz (M3). In the x-direction, only node No. 12648 shows a slight deviation from the others. For material M4, the dominant frequency is the lowest at 4.5 Hz, while for the remaining materials, it is 5.75 Hz. In the y-direction, the dominant response frequency for the analysed nodes is 4.75 Hz for all materials (M1–M7).

A similar analysis was done for the RB2 rock burst. The dominant response frequencies, depending on the material and node, remain within a narrow range of 5.0 Hz (M5, M6, and M7) to 5.75 Hz (M3). The highest dominant frequency of the structural response, 5.75 Hz, was observed at nodes No. 4293 and No. 15459, which correspond to a central node and an edge node located on one of the gable walls of the building. In these nodes, the maximum dominant frequency was calculated for all materials (M1–M7). A dominant frequency of 5.75 Hz was also determined for materials M2 and M3 in nearly all analysed nodes. Conversely, the lowest dominant frequency, 5.0 Hz, was obtained for materials M4–M7 at nodes No. 5567, 6568, and 6579. In the longitudinal direction (y), the dominant frequencies in most nodes are higher than those in the transverse direction (x), reaching approximately 6 Hz. Under RB3 excitation, the dominant frequencies in the transverse direction (x) are greater (5.25 Hz) than those in the longitudinal direction (y, 5.0 Hz). This relationship is consistent across all nodes and materials (M1–M7) and is the reverse of the trend observed under RB2 excitation—comp Table 11.

**Figure 21:**
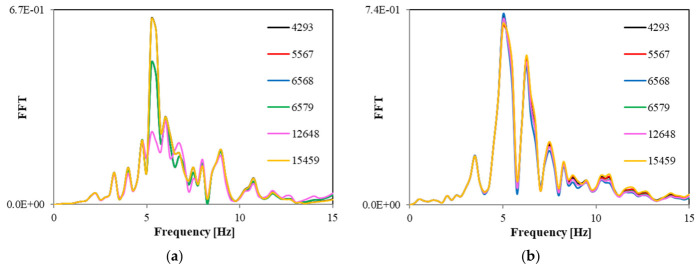
FFT corresponding to response in x (**a**) and y (**b**) directions for material M1 for the RB1 mine-induced tremor based on calculated responses shown in Figure 16.

Based on records from Figure 16, the computed FFT plots for x and y-directions and material M1 for the selected nodes are presented in Figure 21.

The data from Table 8, as well as the FFT curves (presented example graphs from Figure 21 and those for the other material variants and rock bursts), indicate a very small influence of the node location on the determined dominant frequency of vibration accelerations in the time domain for longitudinal direction (y). The calculated dominant frequencies for longitudinal vibration accelerations are practically the same. Only a few shifts in dominant frequency values are observed, whereas in the case of the transverse direction (x), the differences in the dominant vibration acceleration frequencies calculated at various nodes are more noticeable.

## 5. Conclusions

This study presented a comprehensive numerical investigation of the dynamic response of a typical low-rise office building subjected to mining-induced ground vibrations. A validated three-dimensional finite element model (FEM), incorporating soil–structure interaction and recorded ground acceleration time histories, was employed to assess the influence of load-bearing wall materials on structural performance as well as the impact of the choice of the place of numerical estimation on the calculated building dynamic mine-induced response. Based on the findings, the following conclusions can be drawn:

1. The mechanical properties of load-bearing wall materials are a decisive factor in the building’s dynamic response. Structures built with low-stiffness materials, particularly cellular concrete, exhibited significantly higher displacement and acceleration levels compared to those constructed with reinforced concrete or sand–lime bricks.
2. The strongest dynamic responses occurred under the highest-energy mining tremor (RB1), while substantially lower responses were observed for RB2 and RB3. Despite these variations, the relative ranking of wall materials remained consistent, confirming that material characteristics govern structural behaviour more than tremor energy.
3. The building’s dynamic behaviour was strongly direction-dependent. In the longitudinal direction, the response was predominantly modal, influenced by the proximity of excitation frequencies to the structure’s natural frequencies. In contrast, the transverse direction was mainly governed by forced vibration behaviour, driven by the frequency content of the mining-induced ground motion.
4. Dominant frequencies of displacement and acceleration responses were concentrated within a narrow range (approximately 4.5–6.0 Hz) and showed limited sensitivity to node location. This indicates a global response mechanism controlled by overall stiffness and mass distribution rather than local effects.
5. The significant impact of the choice of the place of numerical estimation on the calculated building dynamic mine-induced response (component time history vibrations, peak response values, and resultant vibrations) was observed for all of the discussed material variants of low-rise buildings. This conclusion applies to both displacements and accelerations.
6. The maximum calculated displacements and accelerations at selected nodes demonstrate that assessment of vibration effects on the structure can be adequately performed using a limited number of nodes located in regions of reduced stiffness, i.e., with a lower density of load-bearing walls. A preliminary structural identification of such regions significantly reduces the volume of computational data required for subsequent analyses.
7. The study identifies the minimum number and spatial distribution of measurement points required for the reliable simulation of the dynamic response of this type of low-rise building. The measurement points should be located in regions where the highest dynamic response is expected, i.e., in areas characterised by relatively low stiffness of the structural system. Such locations typically correspond to parts of the building with a low density of load-bearing walls. Consequently, the placement of measurement points in these regions allows the critical dynamic behaviour of the structure to be captured more effectively and improves the accuracy of the numerical simulation results.
8. Among the analysed materials, cellular concrete consistently produced the highest acceleration and displacement amplitudes, reflecting its limited ability to attenuate mining-induced vibrations. Its application in mining-affected regions should therefore be carefully evaluated and supplemented with additional structural measures where necessary.
9. The good agreement between numerical results and in situ measurements confirms the reliability of the adopted FEM approach. Such models provide an effective tool for assessing structural vulnerability, supporting material selection, and informing design and retrofit strategies for buildings exposed to mining-induced vibrations.

A linear-elastic numerical simulation was performed for all considered buildings and rock bursts. It is based on long-term observation of the real structure. Cracking effects and plastic damage of materials were not modelled because the analysed structures are residential buildings, for which even very small damage levels are generally unacceptable to occupants. In mining areas, the occurrence of cracks or other visible defects often results in compensation claims against the mining operator or legal proceedings. Consequently, the adopted approach was aimed at evaluating structural performance within the serviceability range preceding crack development, rather than modelling the cracked state of the structure. Nevertheless, due to the fact that the seismicity of mining areas is constantly increasing, future research directions should take into account the nonlinear approach to long-term cyclic excitations induced by seismic-type mining tremors.

## Figures and Tables

**Table 1:** Construction material parameters of the analysed buildings.

Material	Elastic Modulus [GPa]	Density [kg/m^3^]	Poisson’s Ratio [–]
standard reinforced concrete (M1)	31.0	2500	0.25
high-strength oil palm shell lightweight reinforced concrete (M2)	13.4	1900	0.20
cellular concrete (M3)	1.80	600	0.25
brick (M4)	2.85	1800	0.25
sand–lime brick 1 (M5)	5.56	1820	0.23
sand–lime brick 2 (M6)	6.68	1810	0.21
sand–lime brick 3 (M7)	6.94	1730	0.23

**Table 2:** Rock burst parameters.

Rock Burst	Rock Burst Parameters							
	Energy [J]	Epicentral Distance [m]	Vibration Duration [s]		Peak Horizontal Velocity [m/s]		Main Frequency Intervals [Hz]	
			x	y	x	y	x	y
RB1	8 × 10^7^	899	1.61	1.07	0.031	0.040	8.06–8.55	4.40–5.13
RB2	3 × 10^6^	1408	1.22	1.11	0.009	0.010	14.65–15.14	10.74–11.23
RB3	8 × 10^5^	855	1.01	1.57	0.007	0.005	19.53–21.24	13.43–13.67

**Table 3:** Computed peak displacement values of the building dynamic responses in the considered nodes.

Rock Burst	Material	Direction	Peak Displacement Value [m × 10^−3^]					
			Node 4293	Node 5567	Node 6568	Node 6579	Node 12648	Node 15459
RB1	M1	x	1.005	0.962	0.995	0.939	0.897	0.997
		y	0.827	0.451	0.431	0.446	0.344	0.740
	M2	x	1.063	0.999	1.045	0.976	0.913	1.044
		y	0.918	0.543	0.507	0.532	0.418	0.852
	M3	x	1.223	1.144	1.202	1.124	1.028	1.216
		y	1.153	0.804	0.733	0.773	0.688	1.116
	M4	x	1.202	1.109	1.158	1.090	1.039	1.194
		y	1.121	0.751	0.713	0.735	0.639	1.064
	M5	x	1.149	1.067	1.117	1.045	0.979	1.137
		y	1.039	0.670	0.622	0.654	0.552	0.984
	M6	x	1.129	1.050	1.099	1.028	0.964	1.115
		y	1.006	0.635	0.589	0.620	0.513	0.948
	M7	x	1.119	1.041	1.084	1.020	0.970	1.102
		y	0.969	0.591	0.550	0.578	0.463	0.902
RB2	M1	x	0.167	0.164	0.183	0.163	0.146	0.162
		y	0.233	0.122	0.122	0.123	0.093	0.209
	M2	x	0.176	0.169	0.189	0.167	0.146	0.172
		y	0.257	0.146	0.147	0.148	0.118	0.237
	M3	x	0.202	0.186	0.206	0.185	0.155	0.199
		y	0.314	0.204	0.203	0.205	0.176	0.298
	M4	x	0.219	0.217	0.238	0.215	0.203	0.216
		y	0.307	0.197	0.198	0.200	0.172	0.290
	M5	x	0.197	0.188	0.209	0.187	0.166	0.193
		y	0.288	0.178	0.178	0.180	0.150	0.269
	M6	x	0.192	0.184	0.205	0.183	0.161	0.188
		y	0.280	0.169	0.170	0.171	0.142	0.261
	M7	x	0.194	0.188	0.209	0.187	0.168	0.190
		y	0.271	0.159	0.160	0.161	0.130	0.251
RB3	M1	x	0.077	0.084	0.092	0.083	0.071	0.078
		y	0.096	0.047	0.050	0.050	0.035	0.086
	M2	x	0.082	0.091	0.099	0.090	0.076	0.083
		y	0.107	0.057	0.064	0.062	0.049	0.099
	M3	x	0.092	0.105	0.111	0.105	0.094	0.092
		y	0.138	0.088	0.101	0.096	0.088	0.130
	M4	x	0.090	0.104	0.109	0.103	0.096	0.091
		y	0.125	0.078	0.089	0.085	0.077	0.119
	M5	x	0.087	0.099	0.105	0.098	0.087	0.088
		y	0.120	0.070	0.081	0.077	0.067	0.113
	M6	x	0.086	0.097	0.104	0.096	0.084	0.087
		y	0.116	0.066	0.076	0.073	0.062	0.109
	M7	x	0.085	0.095	0.102	0.095	0.081	0.086
		y	0.110	0.060	0.068	0.066	0.053	0.102

**Table 4:** Amplification coefficients of mean displacements from the considered six nodes.

Rock Burst	Direction	Displacement Amplification Coefficient [–]					
		M2/M1	M3/M1	M4/M1	M5/M1	M6/M1	M7/M1
RB1	x	1.04	1.20	1.17	1.12	1.10	1.09
	y	1.16	1.63	1.55	1.40	1.33	1.25
RB2	x	1.04	1.15	1.33	1.16	1.13	1.15
	y	1.17	1.56	1.52	1.38	1.33	1.26
RB3	x	1.07	1.23	1.22	1.16	1.14	1.12
	y	1.20	1.75	1.57	1.44	1.37	1.25

**Table 5:** Computed resultant peak displacement values of the building’s dynamic response at selected nodes and their corresponding times of occurrence for the RB1 rock burst.

Node No.	M1 (mm; s)	M2 (mm; s)	M3 (mm; s)	M4 (mm; s)	M5 (mm; s)	M6 (mm; s)	M7 (mm; s)
4293	1.259; 0.782	0.261; 0.894	0.317; 0.894	0.312; 0.898	0.292; 0.896	0.284; 0.896	0.275; 0.896
5567	1.008; 0.778	1.066; 0.776	1.262; 0.776	1.260; 0.784	1.173; 0.778	1.146; 0.778	1.130; 0.780
6568	1.023; 0.776	1.079; 0.774	1.261; 0.772	1.263; 0.782	1.179; 0.776	1.154; 0.776	1.139; 0.778
6579	0.091; 0.782	0.103; 0.782	0.133; 0.784	0.124; 0.784	0.117; 0.784	0.113; 0.784	0.108; 0.784
12648	0.912; 0.778	0.948; 0.776	1.103; 0.776	1.156; 0.784	1.052; 0.780	1.025; 0.780	1.022; 0.780
15459	1.177; 0.782	1.283; 0.782	1.543; 0.780	1.541; 0.786	1.434; 0.784	1.397; 0.782	1.370; 0.784

**Table 6:** Vibration dominant frequencies of the building displacement dynamic responses in the considered nodes.

Rock Burst	Material	Direction	Dominant Frequency Value [Hz]					
			Node 4293	Node 5567	Node 6568	Node 6579	Node 12648	Node 15459
RB1	M1	x	5.00	5.00	5.00	5.00	5.00	5.00
		y	4.75	4.75	4.75	4.75	4.75	4.75
	M2	x	5.00	5.00	5.00	5.00	5.00	5.00
		y	4.75	4.75	4.75	4.75	4.75	4.75
	M3	x	5.25	5.00	5.00	5.00	5.00	5.25
		y	4.75	4.75	4.75	4.75	4.75	4.75
	M4	x	5.00	5.00	5.00	5.00	4.50	5.00
		y	4.75	4.75	4.75	4.75	4.75	4.75
	M5	x	5.00	5.00	5.00	5.00	5.00	5.00
		y	4.75	4.75	4.75	4.75	4.75	4.75
	M6	x	5.00	5.00	5.00	5.00	5.00	5.00
		y	4.75	4.75	4.75	4.75	4.75	4.75
	M7	x	5.00	5.00	5.00	5.00	5.00	5.00
		y	4.75	4.75	4.75	4.75	4.75	4.75
RB2	M1	x	5.25	5.25	5.25	5.25	5.00	5.25
		y	6.00	6.00	6.00	6.00	6.00	6.00
	M2	x	5.75	5.25	5.25	5.25	5.00	5.75
		y	6.00	6.00	6.00	6.00	6.00	6.00
	M3	x	5.75	5.25	5.25	5.25	5.00	5.75
		y	6.00	6.00	6.00	6.00	6.00	6.00
	M4	x	5.00	5.00	5.00	5.00	5.00	5.00
		y	5.00	5.00	5.00	5.00	5.00	5.00
	M5	x	5.00	5.00	5.25	5.00	5.00	5.00
		y	5.25	6.00	5.25	6.00	6.00	6.00
	M6	x	5.00	5.00	5.25	5.25	5.00	5.00
		y	6.00	6.00	5.25	6.00	6.00	6.00
	M7	x	5.00	5.00	5.25	5.00	5.00	5.00
		y	5.00	6.00	5.00	5.00	6.00	6.00
RB3	M1	x	5.25	5.25	5.25	5.25	5.25	5.25
		y	5.00	5.00	5.00	5.00	5.00	5.00
	M2	x	5.25	5.25	5.25	5.25	5.25	5.25
		y	5.00	5.00	5.00	5.00	5.00	5.00
	M3	x	5.25	5.25	5.25	5.25	5.25	5.25
		y	5.00	5.00	5.00	5.00	5.00	5.00
	M4	x	5.25	5.25	5.25	5.25	5.25	5.25
		y	5.00	5.00	5.00	5.00	5.00	5.00
	M5	x	5.25	5.25	5.25	5.25	5.25	5.25
		y	5.00	5.00	5.00	5.00	5.00	5.00
	M6	x	5.25	5.25	5.25	5.25	5.25	5.25
		y	5.00	5.00	5.00	5.00	5.00	5.00
	M7	x	5.25	5.25	5.25	5.25	5.25	5.25
		y	5.00	5.00	5.00	5.00	5.00	5.00

**Table 7:** Peak acceleration values of the building dynamic responses in the considered nodes.

Rock Burst	Material	Direction	Peak Acceleration Value [m/s^2^]					
			Node 4293	Node 5567	Node 6568	Node 6579	Node 12648	Node 15459
RB1	M1	x	1.917	1.815	1.806	1.810	1.679	1.918
		y	2.295	2.335	2.097	2.221	2.222	2.439
	M2	x	2.073	2.003	1.994	2.004	1.835	2.071
		y	2.460	2.569	2.263	2.406	2.441	2.653
	M3	x	2.432	2.397	2.386	2.406	2.187	2.427
		y	2.897	3.096	2.663	2.853	2.904	3.165
	M4	x	1.870	1.865	1.853	1.871	1.715	1.871
		y	2.383	2.491	2.241	2.347	2.398	2.565
	M5	x	2.031	1.998	1.988	2.003	1.829	2.029
		y	2.484	2.611	2.308	2.443	2.488	2.685
	M6	x	2.030	1.987	1.976	1.991	1.819	2.026
		y	2.463	2.580	2.286	2.419	2.464	2.661
	M7	x	1.911	1.862	1.854	1.865	1.714	1.914
		y	2.326	2.403	2.168	2.277	2.314	2.493
RB2	M1	x	0.336	0.297	0.298	0.297	0.295	0.335
		y	0.631	0.619	0.592	0.605	0.596	0.661
	M2	x	0.348	0.353	0.352	0.354	0.347	0.347
		y	0.648	0.637	0.618	0.627	0.614	0.678
	M3	x	0.393	0.461	0.457	0.465	0.462	0.387
		y	0.677	0.725	0.644	0.665	0.679	0.744
	M4	x	0.308	0.347	0.345	0.352	0.344	0.308
		y	0.589	0.587	0.566	0.571	0.565	0.627
	M5	x	0.332	0.366	0.365	0.370	0.364	0.331
		y	0.622	0.613	0.600	0.605	0.591	0.652
	M6	x	0.335	0.360	0.359	0.363	0.356	0.334
		y	0.628	0.618	0.604	0.610	0.595	0.658
	M7	x	0.327	0.324	0.324	0.327	0.315	0.326
		y	0.617	0.604	0.590	0.596	0.582	0.652
RB3	M1	x	0.183	0.188	0.189	0.186	0.247	0.176
		y	0.290	0.258	0.301	0.281	0.278	0.304
	M2	x	0.195	0.213	0.211	0.212	0.279	0.187
		y	0.308	0.281	0.330	0.305	0.306	0.327
	M3	x	0.209	0.279	0.273	0.280	0.349	0.204
		y	0.373	0.366	0.395	0.369	0.377	0.406
	M4	x	0.167	0.208	0.204	0.208	0.305	0.161
		y	0.298	0.292	0.317	0.297	0.307	0.323
	M5	x	0.183	0.219	0.215	0.218	0.303	0.177
		y	0.311	0.297	0.334	0.309	0.317	0.335
	M6	x	0.186	0.215	0.213	0.214	0.295	0.179
		y	0.308	0.290	0.330	0.305	0.312	0.329
	M7	x	0.181	0.202	0.200	0.289	0.276	0.173
		y	0.288	0.264	0.309	0.201	0.289	0.306

**Table 8:** Coefficients of variation (CV) and mean values (MV) of the peak acceleration responses in the considered nodes.

Rock Burst	Direction	CV [%]; MV [m/s^2^]						
		M1	M2	M3	M4	M5	M6	M7
RB1	x	4.4; 1.824	4.0; 1.997	3.6; 2.373	3.1; 1.841	3.5; 1.980	3.6; 1.972	3.6; 1.853
	y	4.7; 2.268	5.0; 2.465	5.6; 2.930	4.3; 2.404	4.8; 2.503	4.8; 2.479	4.3; 2.330
RB2	x	5.9; 0.310	0.8; 0.350	7.7; 0.438	5.6; 0.334	4.6; 0.355	3.4; 0.351	1.3; 0.324
	y	3.8; 0.617	3.4; 0.637	5.0; 0.689	3.7; 0.584	3.2; 0.614	3.3; 0.619	3.8; 0.607
RB3	x	12.2; 0.195	13.8; 0.216	18.4; 0.266	22.6; 0.209	18.8; 0.219	17.4; 0.217	20.6; 0.220
	y	5.4; 0.285	5.2; 0.310	3.8; 0.381	3.7; 0.306	4.3; 0.317	4.5; 0.312	13.3; 0.276

**Table 9:** Amplification coefficients of mean accelerations from the considered six nodes.

Rock Burst	Direction	Acceleration Amplification Coefficient [–]					
		M2/M1	M3/M1	M4/M1	M5/M1	M6/M1	M7/M1
RB1	x	1.09	1.30	1.01	1.09	1.08	1.02
	y	1.09	1.29	1.06	1.10	1.09	1.03
RB2	x	1.13	1.41	1.08	1.14	1,13	1.04
	y	1.03	1.12	0.95	0.99	1.00	0.98
RB3	x	1.11	1.36	1.07	1.12	1.11	1.13
	y	1.09	1.34	1.07	1.11	1.10	0.97

**Table 10:** Maximum relevant acceleration (a) and time of occurrence at the selected nodes and materials in the case of the RB1 rock burst.

Node No.	Material	a [m/s^2^]	Time [s]
4293	M1	2.79	0.778
	M2	2.97	0.778
	M3	3.39	0.778
	M4	2.80	0.782
	M5	2.95	0.780
	M6	2.94	0.778
	M7	2.80	0.780
5567	M1	2.68	0.778
	M2	2.87	0.778
	M3	3.32	0.780
	M4	2.74	0.782
	M5	2.88	0.780
	M6	2.86	0.780
	M7	2.71	0.780
6568	M1	2.47	0.778
	M2	2.59	0.778
	M3	2.91	0.780
	M4	2.51	0.784
	M5	2.59	0.782
	M6	2.58	0.780
	M7	2.49	0.782
6579	M1	2.57	0.778
	M2	2.71	0.778
	M3	3.09	0.780
	M4	2.60	0.784
	M5	2.71	0.782
	M6	2.70	0.780
	M7	2.59	0.782
12648	M1	2.62	0.776
	M2	2.54	0.780
	M3	2.94	0.784
	M4	2.51	0.784
	M5	2.58	0.782
	M6	2.56	0.782
	M7	2.44	0.782
15459	M1	2.92	0.778
	M2	3.12	0.778
	M3	3.60	0.780
	M4	2.96	0.784
	M5	3.11	0.780
	M6	3.10	0.780
	M7	2.94	0.780

**Table 11:** Vibration dominant frequencies of the building acceleration dynamic responses in the considered nodes.

Rock Burst	Material	Direction	Dominant Frequency Value [Hz]					
			Node 4293	Node 5567	Node 6568	Node 6579	Node 12648	Node 15459
RB1	M1	x	5.00	5.00	5.00	5.00	5.75	5.00
		y	4.75	4.75	4.75	4.75	4.75	4.75
	M2	x	5.00	5.00	5.00	5.00	5.75	5.00
		y	4.75	4.75	4.75	4.75	4.75	4.75
	M3	x	5.25	5.25	5.25	5.25	5.75	5.25
		y	4.75	4.75	4.75	4.75	4.75	4.75
	M4	x	5.00	5.00	5.00	5.00	4.50	5.00
		y	5.00	5.00	5.00	5.00	5.00	5.00
	M5	x	5.00	5.00	5.00	5.00	5.75	5.00
		y	4.75	4.75	4.75	4.75	4.75	4.75
	M6	x	5.00	5.00	5.00	5.00	5.75	5.00
		y	4.75	4.75	4.75	4.75	4.75	4.75
	M7	x	5.00	5.00	5.00	5.00	5.75	5.00
		y	4.75	4.75	4.75	4.75	4.75	4.75
RB2	M1	x	5.75	5.25	5.25	5.25	5.25	5.75
		y	6.00	6.00	5.25	6.00	6.00	6.00
	M2	x	5.75	5.75	5.75	5.75	5.25	5.75
		y	6.00	6.00	6.00	6.00	6.00	6.00
	M3	x	5.75	5.75	5.75	5.75	5.25	5.75
		y	6.00	6.00	6.00	6.00	6.00	6.00
	M4	x	5.75	5.00	5.00	5.00	5.25	5.75
		y	5.00	5.00	5.00	5.00	5.00	5.00
	M5	x	5.75	5.00	5.00	5.00	5.25	5.75
		y	6.00	5.75	5.25	6.00	6.00	5.75
	M6	x	5.75	5.00	5.00	5.00	5.25	5.75
		y	6.00	5.75	5.25	6.00	6.00	6.00
	M7	x	5.75	5.00	5.00	5.00	5.25	5.75
		y	5.25	5.75	5.25	5.25	6.00	5.75
RB3	M1	x	5.25	5.25	5.25	5.25	5.25	5.25
		y	5.00	5.00	5.00	5.00	5.00	5.00
	M2	x	5.25	5.25	5.25	5.25	5.25	5.25
		y	5.00	5.00	5.00	5.00	5.00	5.00
	M3	x	5.25	5.25	5.25	5.25	5.25	5.25
		y	5.00	5.00	5.00	5.00	5.00	5.00
	M4	x	4.75	4.75	5.25	5.25	5.25	4.75
		y	5.00	5.00	5.00	5.00	5.00	5.00
	M5	x	5.25	5.25	5.25	5.25	5.25	5.25
		y	5.00	5.00	5.00	5.00	5.00	5.00
	M6	x	5.25	5.25	5.25	5.25	5.25	5.25
		y	5.00	5.00	5.00	5.00	5.00	5.00
	M7	x	5.25	5.25	5.25	5.25	5.25	5.25
		y	5.00	5.00	5.00	5.00	5.00	5.00

## Data Availability

The original contributions presented in this study are included in the article. Further inquiries can be directed to the corresponding author.

## References

[1] Zembaty Z. (2004). Rockburst induced ground motion—A comparative study. Soil Dyn. Earthq. Eng..

[2] Pachla F., Tatara T., Köber D., De Stefano M., Zembaty Z. (2020). Dynamic Resistance of Residential Masonry Building with Structural Irregularities. Seismic Behaviour and Design of Irregular and Complex Civil Structures III. Geotechnical, Geological and Earthquake Engineering.

[3] Zajac M., Kuzniar K., Tatara T. (2025). Influence of load-bearing wall material properties on building mine-induced dynamic response. Sci. Rep..

[4] Zajac M., Kuzniar K., Tatara T. (2024). Influence of Subsoil and Building Material Properties on Mine-Induced Soil-Structure Interaction Effect. Appl. Sci..

[5] Zajac M., Kuzniar K., Tatara T. (2024). Effect of Load-Bearing Wall Material on Building Dynamic Properties. Materials.

[6] Sołtys A., Pyra J. (2024). The Influence of Vibrations Induced by Blasting Works in an Open-Pit Mine and Seismic Events in an Underground Mine on Building Structures—A Case Study. Appl. Sci..

[7] Tiwari S., Adhikari S. (2023). Seismic Behaviour of the Low-Rise RC Buildings in Nonlinear Static and Dynamic Analysis. Saudi J. Civ. Eng..

[8] Dedeoğlu I.Ö., Yetkin M., Calayır Y. (2024). Seismic performance of masonry structures in the rural area during the November 23, 2022, Düzce-Gölyaka earthquake. J. Build. Eng..

[9] Okur E.K., Altunışık A.C., Okur F.Y., Meral M., Yilmaz Z., Günaydin M., Genç A.F. (2021). Dynamic response of a traditional hımış mansion using updated FE model with operational modal testing. J. Build. Eng..

[10] Askouni P.K., Karabalis D.L. (2022). The Modification of the Estimated Seismic Behaviour of R/C Low-Rise Buildings Due to SSI. Buildings.

[11] Dutta S.C., Bhattacharya K., Roy R. (2004). Response of low-rise buildings under seismic ground excitation incorporating soil–structure interaction. Soil Dyn. Earthq. Eng..

[12] (2016). Evaluation of Harmfulness of Vibrations Transmitted Through the Ground to Buildings.

[13] Vieille J., Negulescu C., Gehl P., Dominique P., Guidez R., Mutke G. (2025). Damage assessment in the case of post-mining-induced seismicity: How to use and adapt macroseismic intensity scales?. J. Sustain. Min..

[14] Kuzniar K. (2011). Neural networks for the analysis of mine-induced building vibrations. Comput. Assist. Methods Eng. Sci..

[15] Zajac M.C., Kuzniar K. (2024). Convolutional Neural Networks in the SSI Analysis for Mine-Induced Vibrations. Comput. Assist. Methods Eng. Sci..

[16] Lizarazo-Marriaga J., Vargas C.A., Tiria L. (2018). A new approach to predict local site effects related to blast-induced ground vibrations. J. Geophys. Eng..

[17] Gong M., Liu B., Wang X., Zhou B., Zhao Y. (2025). Damage assessment of reinforced concrete frame under mainshock-aftershock based on deep learning considering pre-earthquake damage. J. Build. Eng..

[18] Jiao C., You S., Ji H. (2023). Numerical analysis of building structures response under effect of continuous and stepped non-uniform settlement. J. Cent. South Univ..

[19] Hrubesova E., Kalab Z. Modeling of the Mining Induced Seismicity Impact on the Building Using Numerical System Plaxis. Proceedings of the ISRM International Symposium EUROCK 2005.

[20] Kallioras S., Graziotti F., Penna A. (2019). Numerical assessment of the dynamic response of a URM terraced house exposed to induced seismicity. Bull. Earthq. Eng..

[21] Bu H., Jiang H., Sanada Y. (2025). A unified simplified numerical model for large-scale regional seismic simulation of building structures. J. Build. Eng..

[22] Requena-Garcia-Cruz M.V., Romero-Sánchez E., Morales-Esteban A. (2025). Contribution of the soil-structure interaction to the seismic behaviour of the Mosque-Cathedral of Córdoba. J. Build. Eng..

[23] Maciag E., Kuzniar K., Tatara T. (2016). Response Spectra of Ground Motions and Building Foundation Vibrations Excited by Rockbursts in the LGC Region. Earthq. Spectra.

[24] ANSYS (2017). Ansys Mechanical APDL Structural Analysis Guide.

[25] Lipinski J. (1985). Machine Foundation.

[26] Lysmer J., Kuhlemeyer R.L. (1969). Finite Dynamic Model for Infinite Media. J. Eng. Mech. Div..

[27] Lysmer J., Richart F.E. (1966). Dynamic Response of Footings to Vertical Loading. J. Soil Mech. Found. Div..

[28] Far H. (2019). Advanced computation methods for soil-structure interaction analysis of structures resting on soft soils. Int. J. Geotech. Eng..

[29] Wolf J. (1994). Foundation Vibration Analysis Using Simple Physical Models.

[30] (2004). Eurocode 8: Design of Structures for Earthquake Resistance. Part 1: General Rules, Seismic Actions and Rules for Buildings.

[31] Papagiannopoulos G.A., Beskos D.E. (2006). On a modal damping identification model of building structures. Arch. Appl. Mech..

[32] Kuzniar K., Tatara T., Zajac M. (2024). Experimental and numerical assessment of soil-structure interaction effects in the case of mine-induced vibrations. J. Phys. Conf. Ser..

[33] Chopra A.K. (2012). Dynamics of Structures: Theory and Applications to Earthquake Engineering.

